# Sialic Acid Is Required for Neuronal Inhibition by Soluble MAG but not for Membrane Bound MAG

**DOI:** 10.3389/fnmol.2016.00021

**Published:** 2016-04-01

**Authors:** Najat Al-Bashir, Wilfredo Mellado, Marie T. Filbin

**Affiliations:** ^1^Biology Department, Hunter College, City University of New YorkNew York, NY, USA; ^2^Burke-Cornell Medical Research Institute White Plains, NY, USA

**Keywords:** myelin inhibition, MAG, gangliosides, sialic acid, neurite outgrowth

## Abstract

Myelin-Associated Glycoprotein (MAG), a major inhibitor of axonal growth, is a member of the immunoglobulin (Ig) super-family. Importantly, MAG (also known as Siglec-4) is a member of the Siglec family of proteins (sialic acid-binding, immunoglobulin-like lectins), MAG binds to complex gangliosides, specifically GD1a and/or GT1b. Therefore, it has been proposed as neuronal receptors for MAG inhibitory effect of axonal growth. Previously, we showed that MAG binds sialic acid through domain 1 at Arg118 and is able to inhibit axonal growth through domain 5. We developed a neurite outgrowth (NOG) assay, in which both wild type MAG and mutated MAG (MAG Arg118) are expressed on cells. In addition we also developed a soluble form NOG in which we utilized soluble MAG-Fc and mutated MAG (Arg118-Fc). Only MAG-Fc is able to inhibit NOG, but not mutated MAG (Arg118)-Fc that has been mutated at its sialic acid binding site. However, both forms of membrane bound MAG- and MAG (Arg118)- expressing cells still inhibit NOG. Here, we review various results from different groups regarding MAG’s inhibition of axonal growth. Also, we propose a model in which the sialic acid binding is not necessary for the inhibition induced by the membrane form of MAG, but it is necessary for the soluble form of MAG. This finding highlights the importance of understanding the different mechanisms by which MAG inhibits NOG in both the soluble fragmented form and the membrane-bound form in myelin debris following CNS damage.

## Introduction

**Figure 1:**
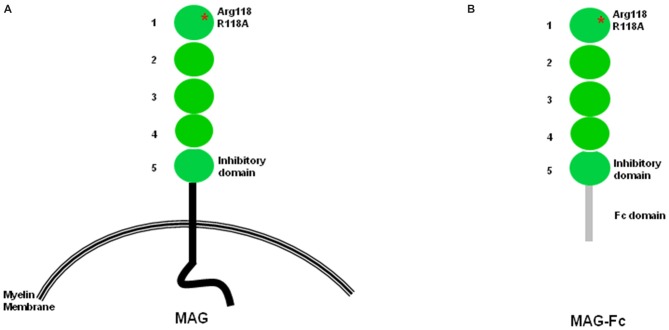
**Structural features of myelin-associated glycoprotein (MAG). (A)** Structural features of MAG. Arginine 118, the sialic acid binding site and also part of the RGD site, is located within domain 1. The inhibition site is located within domain 5. **(B)** In soluble MAG-Fc, the protein portion after domain 5 is replaced by the Fc domain.

Myelin-associated glycoprotein (MAG), a member of the immunoglobulin (Ig) super-family, contains five Ig-like domains in its extracellular sequences, a single transmembrane domain, and a short cytoplasmic domain (Figure 1A; Lai et al., 1987a,b; Salzer et al., 1987, 1990). Since its recognition as a potent inhibitor of central nervous system (CNS) axon regeneration (McKerracher et al., 1994; Mukhopadhyay et al., 1994), enormous efforts have been directed at identifying its receptors. MAG was first reported as a sialic acid binding protein that interacts with the complex gangliosides GT1b and GD1a (Vinson et al., 2001). The search for receptors that transduce its inhibitory effect resulted in the discovery of NgR1, NgR2, and PirB as functional MAG receptors (Domeniconi et al., 2002; Liu et al., 2002; Venkatesh et al., 2005; Atwal et al., 2008). MAG was also shown to bind and signal through β-1 integrin (Goh et al., 2008). Recently, the inhibition of neurite outgrowth (NOG) of cortical neurons plated on MAG-expressing Chinese hamster ovary (MAG-CHO) cells was found to be independent of NgRs and gangliosides. Rather, PTEN, a lipid phosphatase that activates AKT, was found to mediate the MAG inhibitory signal, suggesting the existence of other, as yet unknown, receptors (Perdigoto et al., 2011).

Discovering MAG receptors is critical for identifying therapeutic targets for promoting axonal regeneration, and it is also important to clarify the role of gangliosides in MAG-induced inhibition of NOG. Here, we focus on the relationship between MAG and sialic acid in gangliosides, please see a recent extensive review of sialic acids (Schnaar et al., 2014).

## MAG as a Member of the Siglec Protein Family

The Siglec protein family is a subgroup of the Ig super-family that has similar amino acid sequences in the first four Ig-like domains and shows sialic acid-dependent binding to cells (Kelm et al., 1998). Members include sialoadhesin (Siglec-1), CD22 (Siglec-2), CD33 (Siglec-3), and MAG (Siglec-4; DeBellard et al., 1996; Tang et al., 1997a; Kelm et al., 1998). Each member of the Siglec family binds with different specificity. MAG preferentially binds α2,3-linked sialic acid residues attached to O-linked glycoconjugates (Kelm et al., 1994), CD22 binds to α2,6-linked sialic acid attached to N-linked glycoconjugates, and sialoadhesin and CD33 recognize α2,3-linked sialic acid attached to O- or N-linked glycoconjugates (Kelm et al., 1994; Cornish et al., 1998).

MAG is expressed early in development (for a thorough description of MAG in normal tissue, see Baldwin and Giger, 2015) exclusively at the interface between myelinated axons and periaxonal myelin membrane (Trapp et al., 1989). MAG plays a role in axon-glial interactions (Schachner and Bartsch, 2000) and influences myelin formation. Genetic depletion of MAG results in altered myelination, reduced axon caliber, reduced neurofilament spacing and phosphorylation, and progressive axonal degeneration (Li et al., 1994; Montag et al., 1994; Fruttiger et al., 1995; Yin et al., 1998).

Early postnatal MAG-deficient mice exhibit delays in the formation of compact myelin in the optic nerves and reduced density of retinal ganglion cell axons surrounded by compact myelin (Montag et al., 1994). Although the ultrastructure of compact myelin is unaffected, MAG-deficient mice exhibit a dilated periaxonal space and abnormal formation of the periaxonal cytoplasmic collar (Li et al., 1994; Montag et al., 1994). Some axons in MAG-deficient mice are surrounded by more than one myelin sheath (Bartsch, 1996). Moreover, in adult MAG-deficient mice, oligodendrocytes show degeneration of distal processes in the periaxonal space or within compact myelin, which is also observed in immune-mediated demyelinating diseases including multiple sclerosis (Rodriguez-Peña et al., 1993; Lassmann et al., 1997). Thus, in the CNS, MAG is involved in the initiation of myelination, formation of myelin sheaths, and long-term maintenance of oligodendrocyte structure and myelin integrity. In the peripheral nervous system (PNS), MAG seems to be involved only in the formation of intact myelin and long-term maintenance of myelin structure but not in the initiation of myelination.

Collectively, therefore, *in vitro* experiments and studies using MAG-deficient mice show that MAG is a cell adhesion molecule, a receptor that transduces signals into the interior of myelin-forming glial cells, and a contributor to cross-talk between myelin-forming glial cells and axons.

## Inhibitory Site on MAG

Using several chimeric constructs in which domains 4 and 5 of MAG are exchanged with the corresponding domains of sialoadhesin, the Filbin lab showed that the MAG inhibition site is on domain Ig-5 (Figure 1) and is distinct from the sialic acid binding site on domain Ig-1 (Cao et al., 2007). Importantly, several chimeric molecules containing the sialic acid binding site sialoadhesin do not inhibit NOG, reinforcing the notion that the sialic acid binding domain is not necessary for neuronal inhibition (Cao et al., 2007). Another group arrived at a similar conclusion using a different set of molecular tools focusing on domain Ig-4 of neural cell adhesion molecule (N-CAM) and domain Ig-5 of MOG (Wörter et al., 2009).

## Sialic Acid as Component of Gangliosides

Gangliosides are glycosphingolipids containing one or more sialic acid residues in their oligosaccharide structure (Sonnino et al., 2007). They are components of all animal cell membranes and are particularly abundant in the plasma membranes of neurons. Gangliosides are complex lipids with a strong, amphiphilic, big saccharide head-group and a double-tailed hydrophobic moiety. The lipid moiety of gangliosides, shared across sphingolipids, is called a ceramide and is constituted by a long-chain amino alcohol termed sphingosine (Karlsson, 1970), connected to fatty acids by an amide linkage. Sialic acid is a sugar that differentiates gangliosides from neutral glycosphingolipids and sulfatides and defines all derivatives of 5-amino-3,5-dideoxy-d-glycero-d-galacto-non-2-ulopyranosonic acid or neuraminic acid (Schauer, 1982). Gangliosides are positioned to interact with other molecules in their own membrane and molecules on opposing cell membranes (Lopez and Schnaar, 2009). Gangliosides are typically anchored in the outer leaflet of the plasma membrane, where they are driven by ceramide to partition laterally into lipid rafts, which are membrane micro-domains containing other sphingolipids, cholesterol, and signaling molecules. This lateral interaction in the membrane normally results in ganglioside-mediated regulation of membrane proteins, such as receptor kinases. Ganglioside glycans also extend outward from the cell surface, where their sialoglycans participate in intermolecular interactions. This interaction with proteins on opposing membranes results in ganglioside-mediated cell-cell recognition, such as myelin-axon interaction.

**Figure 2:**
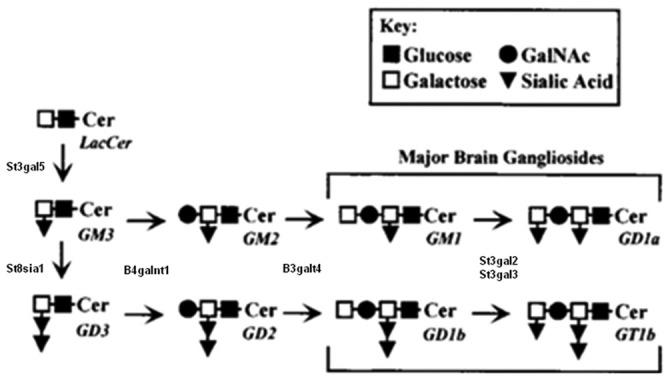
**Partial biosynthesis pathway for major brain gangliosides.** Schematic biosynthetic relationship between major brain gangliosides and their precursors. Disruption of the *B4galnt1* gene blocks the synthesis of gangliosides, including GT1b and GD1a, to which MAG binds. *B4galnt1*-deficient mice lack all complex gangliosides but express higher levels of simple gangliosides GM3 and GD3. Modified from Sheikh et al. (1999), reproduced with permission.

Ceramide is the common precursor of glycosphingolipids and sphingomyelin and is transported from the endoplasmic reticulum to the Golgi apparatus by unknown mechanisms. Glycosphingolipids are synthesized by the stepwise addition of monosaccharide sugars to ceramide and the growing sugar. Subsequent addition of galactose, sialic acid, and N-acetylgalactosamine from their nucleotide sugar donors to the growing saccharide chain generates penta, hexa, and hepta saccharide glycans (Kolter et al., 2002). The ganglioside biosynthetic pathway (Figure 2) involves a sequential process of glycosylation via two main pathways: the “a” series (GM2, GM1a, GD1a) and “b” series (GD2, GD1b, GT1b; van Echten and Sandhoff, 1993). Each ganglioside is structurally more complex than its precursor molecule, and the stepwise addition of monosaccharide or sialic acid residues by specific membrane-bound glycosyltransferases in the Golgi apparatus is catalyzed by the same glycosyltransferases in both pathways. In an investigation of the differential distribution of GM1, GD1a, GD1b, and GT1b in the adult mouse CNS (Vajn et al., 2013), GD1b and GT1b was expressed in gray and white matter, GM1 was expressed in white matter, and GD1a was expressed in specific nuclei/tracts. This differential expression of gangliosides could explain why MAG appears to use different receptors on different neurons to inhibit NOG (Mehta et al., 2007; Venkatesh et al., 2007).

## B4GALNT1-Deficient Mice

Among many glycosyltransferases, β1, 4 GalNAc-transferase (β1, 4 GalNAc-T; GM2/GD2 synthase; EC2.4.1.92), coded by the gene B4GALNT1, plays an important role in the biosynthesis of almost all complex gangliosides (Figure 2). Two different groups independently disrupted the B4GALNT1 gene to generate *B4galnt1*-deficient mice. Takamiya et al. (1996) disrupted exon 4 of the B4GALNT1 gene by inserting a neomycin-resistant plasmid and found that *B4galnt1*-deficient mice express no complex gangliosides but higher concentrations of the simple gangliosides GD3 and GM3. By 12 weeks of age, *B4galnt1*-deficient mice show decreased central conduction velocity but normal brain weight and shape, myelination of white matter, and myelinated fiber and synapse structure, suggesting that complex gangliosides are required for neuronal function but not brain organogenesis. This lack of complex gangliosides might be compensated by higher levels of GM3 and GD3 in these mice. A later study reports that deficits exhibited by these *B4galnt1*-deficient mice can be rescued by tissue-specific GalNacT constructs. Using the neurofilament light promoter (restricted to neurons) and proteolipid protein promoter (restricted to myelinating glia, including oligodendrocytes and Schwann cells), the restoration of complex gangliosides in neurons but not glia was found to be critical for maintaining CNS integrity (Yao et al., 2014).

Sheikh et al. (1999) generated a different strain of *B4galnt1*-deficient mice using a vector, in which exon 6 and 7 and part of exon 8 are deleted and replaced with a MC1NeoPolyA selection cassette (Sheikh et al., 1999). These mice have a normal life span but show decreased central myelination, central and peripheral axonal degeneration, and increased levels of GD3 and GM3. Similar to MAG-deficient mice, *B4galnt1*-deficient mice show abnormalities, such as doubly myelinated axons with cytoplasm between the two myelin sheaths. Whereas young *B4galnt1*-deficient mice express normal levels of MAG, adult *B4galnt1*-deficient mice show a ~30% decline in MAG levels in the CNS and PNS and develop Wallerian degeneration (Sheikh et al., 1999). Sheikh et al. (1999) attribute the differences between their findings and the findings of Takamiya et al. (1996) to the fact that the first group’s animals were examined at a young age, whereas the effects of *B4galnt1* deficiency become more prominent with increasing age. They argue that because conduction velocity depends on myelination and axon diameter, the decreased conduction velocity observed by the first group could be attributed to myelination defects and axonal atrophy. Indeed, in a later study, MAG protein but not mRNA was found to decrease substantially by 12 months of age (Sun et al., 2004).

The more recently generated double-heterozygous mice carry null mutations in B4GALNT1 (called *Galgt1*) and ST3GAL5 (called *Siat9*, which encodes GM3 synthase [CMP-NeuAc:lactosylceramide α-2,3-sialytransferase; EC 2.4.99.9]; Yamashita et al., 2005). Although the mice are viable, they exhibit neurodegeneration with severe pathology in white matter and CNS axons. In addition, the mice were generated with mutated ST3GAL2 and ST3GAL3, which are sialyltrasferase genes responsible for terminal sialylation of gangliosides and biosynthesis of GD1a and GT1b (Figure 2). These mice exhibit dysmyelination marked by substantial reductions in major proteins, myelinated axons, myelin thickness, and MAG expression, as well as molecular defects in the Nodes of Ranvier (Yoo et al., 2015), indicating that terminal sialylation is required for optimal brain structure and function.

## Role of Gangliosides in MAG-Mediated Inhibition of NOG

In the NOG assay developed by the Filbin lab, primary neurons are grown on a monolayer of control CHO or MAG-CHO cells, and the growth of neurites is quantified 24 h later (Mukhopadhyay et al., 1994). In this assay, MAG inhibits the growth of postnatal neurons (Mukhopadhyay et al., 1994; DeBellard et al., 1996). Also, removal of neuronal sialic acid residues by sialidase treatment does not alter the inhibitory effect of MAG on NOG (Tang et al., 1997a). The sialic acid binding site on MAG was mapped at Arginine (Arg) 118, a highly conserved residue in many Siglecs, which is part of a RGD site that binds and signals through β-1 integrin (Goh et al., 2008). Arg 118 is located in the first Ig-like domain. Surprisingly, mutations of this amino acid to either Alanine or Aspartate do not affect the inhibitory effect of MAG (Tang et al., 1997a), indicating that MAG-sialic acid interactions might not be required for MAG inhibition.

The Filbin lab also used purified soluble forms of the extracellular domain of MAG fused to the Fc portion (MAG-Fc; Figure 1B) of human IgG1 (Kelm et al., 1994; Tang et al., 1997b). In this modified, soluble-NOG assay, neurons are incubated with MAG-Fc and then plated on L1-Fc substrate in wells coated with anti-Fc antibodies. Although MAG-Fc inhibits the outgrowth of neurites, no inhibition occurs when mutated Fc constructs (MAG (R118A)-Fc or MAG (R118D)-Fc) are used (Tang et al., 1997a), suggesting that MAG-sialic acid interactions are necessary only when MAG is in soluble form. In addition, a truncated form of MAG containing the first three Ig-like MAG domains (MAG d1–3-Fc), which binds neurons in a sialic acid-dependent manner, does not inhibit neurite growth in the soluble-NOG assay (Tang et al., 1997a).

Similarly, Vinson et al. (2001) report that mutation of the R118 residue in the first Ig-like domain reduces the potency of MAG inhibition in the soluble-NOG assay. MAG specifically binds both GT1b and GD1a, which are expressed at the surface of MAG-responsive neurons. Pre-incubation of hippocampal neurons (HNs) with increasing concentrations of alpha methyl sialic acid 3 sialyllactose before addition of MAG-Fc blocks MAG-Fc inhibition of NOG. Also, pre-incubation of HNs with purified GT1b and GD1a blocks MAG- and MAG (R118A)-Fc inhibition, indicating that MAG-GT1b interactions on the cell surface may be a mechanism of MAG-induced inhibition of NOG (Vinson et al., 2001).

Vyas et al. (2002) report that GD1a and GT1b are functional nerve cell ligands for MAG. Treatment of cerebellar granular neurons (CGNs) with *V. cholerae* neuraminidase, which removes sialic acid from cell surfaces, reverses MAG inhibition. Under the same conditions, depletion of gangliosides using the glycosylceramide synthase inhibitor P4 reverses MAG inhibition of NOG. Also, CGNs from mice lacking the complex gangliosides GD1a and GT1b (i.e., *B4galnt1*-deficient mice) are less vulnerable to MAG inhibition. Furthermore, experiments where CGNs are incubated on detergent-extracted myelin or membrane purified from MAG-CHO cells provide additional evidence of the importance of complex gangliosides when MAG is expressed in soluble form.

The Filbin lab also showed that soluble MAG released from damaged white matter, consisting of a proteolytic MAG fragment containing the entire extracellular domain (d1–5), inhibits axonal regeneration (Tang et al., 2001). This finding highlights the importance of understanding the different mechanisms by which MAG inhibits NOG in both the soluble fragmented form and the membrane-bound form in myelin debris following CNS damage.

An interesting experiment by Vinson et al. (2001) sheds light on the function of sialic acid. They demonstrated that aggregating sialic acid, achieved by adding IgM generated from anti-GT1b antibodies, reduces NOG in dose-dependent manner (Vinson et al., 2001), mimicking the inhibitory effect of MAG. Also, pre-incubation of GT1b antibody with purified GT1b, but not with other purified gangliosides, blocks inhibition of NOG by anti-GT1b antibodies. Finally, a Rho kinase inhibitor blocks inhibition of NOG by MAG or anti-GT1b antibody in both HNs and CGNs (Vinson et al., 2001). By contrast, Vyas and colleagues show that highly multivalent clustering of either anti-GT1b or anti-GD1a, achieved using IgG1 monoclonal antibody pre-incubated with secondary anti-Fc, mimics MAG-mediated inhibition of NOG (Vinson et al., 2001; Vyas et al., 2002). This inhibition cannot be due to gangliosides, which do not have transmembrane properties, but rather to another molecule that crosses the membrane and perhaps interacts with gangliosides.

Indeed, lipid rafts have been suggested as a possible transducer of MAG inhibitory signals (McKerracher, 2002). One study in particular indicates that myelin rafts may associate with neurons (Vinson et al., 2003). Specifically, MAG is found in a section of the membrane characterized by its solubility to Triton X-100 but not to Lubrol WX. MAG-Fc detected this section in lipid raft preparations from CGNs in a region containing GT1b, p75, Rho, NgR, and caveolin (Vinson et al., 2003). In another study, MAG-Fc, anti-GD1a, or anti-GT1b brings p75 (and also Flotilin-1, a marker of lipid rafts) to the Brij-58 insoluble lipid raft fraction isolated from mouse CGNs (Fujitani et al., 2005). Also, biochemical studies show the binding of GT1b and GM1 to NgR1 (Williams et al., 2008). Analytical ultracentrifugation analysis shows that GT1b and GM1, but not asialo-GM1, induce additional peaks in a concentration-dependent manner, with higher sedimentation coefficients than the coefficient of NgR1(310)-Fc. When the GT1b binding site on NgR1 is blocked with a cyclic peptide, GT1b antibody fails to inhibit NOG. Finally, GT1b antibody does not inhibit NOG in neurons from NgR1 knock-out mice (Williams et al., 2008). Together, these findings indicate that the effect of GT1b antibody requires NgR1.

## Conclusions: A Working Model

**Figure 3:**
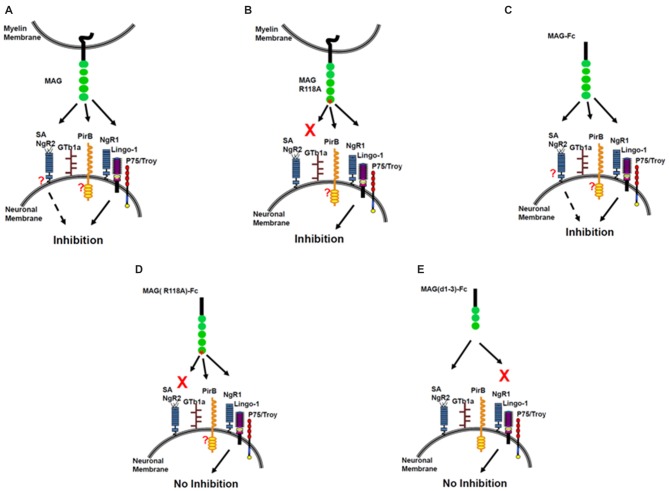
**The sialic acid binding on MAG R118 is critical for inhibition of axonal regeneration by soluble MAG-Fc but not by MAG expressed by Chinese hamster ovary (CHO) cells.** Inhibition of NOG by MAG-CHO **(A)**, mutated MAG (R118A)-CHO **(B)** and soluble MAG-Fc **(C)**. However, mutated soluble MAG (R118A)-Fc does not inhibit NOG **(D)** nor, a truncated form of soluble MAG (d1–3)-Fc, that binds to sialic acid but lacks the inhibitory domain of MAG **(E)**.

In summary, MAG binds sialic acid at Arg118, which is located in domain 1 of MAG, distant from the inhibitory site in domain 5. Soluble MAG requires sialic acid binding for inhibition of NOG. When Arg118 is mutated, inhibition by soluble MAG is abolished. However, sialic acid binding is not required for MAG-expressing CHO cells to inhibit NOG. We conclude that there are at least two binding sites on MAG—the sialic acid binding site at Arg 118 and inhibitory site at domain 5. Therefore, we propose a two-site model for MAG inhibition (Figure 3). When CHO cells express MAG, both sites engage neurons, and NOG is inhibited (Figure 3A). When CHO cells express MAG that is mutated at its sialic acid binding site, NOG is still inhibited (Figure 3B). However, it is unclear whether another molecule helps achieve the correct orientation for inhibition to occur and/or allow membrane-associated MAG to achieve the correct conformation to interact with the receptor. When soluble MAG is added to neurons, both sites engage neurons, and NOG is inhibited (Figure 3C). By contrast, when soluble MAG is mutated at the sialic acid binding site, it cannot bind to neurons, and the inhibition site cannot be engaged, resulting in no inhibition of NOG (Figure 3D). When soluble MAG (d1–3) is added to neurons, it binds through its sialic acid binding site but does not inhibit axonal growth because the inhibition site is absent (Figure 3E). This model suggests that gangliosides are necessary in order to inhibit NOG, but only when soluble form of MAG is present; perhaps gangliosides help achieve correct alignment to NgR, but not when MAG is expressed by CHO cells.

## Author Contributions

NA-B and WM wrote the manuscript, made direct contributions to the work, and approved it for publication. Work in the lab was supported by the Specialized Neuroscience Research Program (NINDS Grants 3U54NS041073 and RO1NS037060) and an infrastructure grant from the Research Centers in Minority Institutions Program (NCRR Grant RR003037) at Hunter College. This work is dedicated to the memory of Marie T. Filbin.

## Conflict of Interest Statement

The authors declare that the research was conducted in the absence of any commercial or financial relationships that could be construed as a potential conflict of interest.
